# Walking with increasing acceleration is achieved by tuning ankle torque onset timing and rate of torque development

**DOI:** 10.1098/rsif.2022.0035

**Published:** 2022-06-29

**Authors:** Logan Wade, Jonathon Birch, Dominic James Farris

**Affiliations:** ^1^ Department for Health, University of Bath, Bath, UK; ^2^ Centre for Analysis of Motion, Entertainment Research and Applications, University of Bath, Bath, UK; ^3^ School of Human Movement and Nutrition Sciences, The University of Queensland, Brisbane, Queensland, Australia; ^4^ Sport and Health Sciences, University of Exeter, Exeter, UK

**Keywords:** constant speed, gait, torque–angle relationship, exoskeleton, prosthetics, falling

## Abstract

Understanding the mechanics of torque production about the ankle during accelerative gait is key to designing effective clinical and rehabilitation practices, along with developing functional robotics and wearable assistive technologies. We aimed to explore how torque and work about the ankle is produced as walking acceleration increases from 0 to 100% maximal acceleration. We hypothesized that as acceleration increased, greater work about the ankle would not be solely due to ramping up plantar flexor torque, and instead would be a product of adjustments to relative timing of ankle torque and angular displacement. Fifteen healthy participants performed walking without acceleration (constant speed), as well as low, moderate and maximal accelerations, while motion capture and ground reaction force data were recorded. We employed vector coding in a novel application to overcome limitations of previously employed evaluation methods. As walking acceleration increased, there was reduced negative work and increased positive work about the ankle. Furthermore, early stance dorsiflexion had reducing plantar flexor torque due to delayed plantar flexor torque onset as acceleration increased, while mid-stance ankle plantar flexor torque was substantially increased with minimal ankle dorsiflexion, irrespective of acceleration magnitude. Assistive devices need to account for these changes during accelerative walking to facilitate functional gait.

## 1. Introduction

Understanding the mechanics of healthy human gait is key to designing effective clinical treatments and rehabilitation practices [1], in addition to the development of robotics and wearable assistive technologies [2–4]. While research has primarily focussed on walking at constant speeds [5–7], 40% of walking bouts are performed with less than 12 steps [8]. As such, accelerations are a crucial component of ambulation, and it is important to understand how altered joint coordination strategies are adjusted to produce accelerative gait.

Accelerative walking is typically associated with production of positive network produced about the ankle during stance, while constant speed walking ranges between zero or slightly positive network output about the ankle [5,9–11], which increases at faster walking speeds [12]. During preferred constant speed walking, ankle plantar flexor muscles remain relatively isometric and work is primarily performed by storing and returning energy within the Achilles tendon [13]. Alternatively, accelerative gait is associated with increased ankle dorsiflexion at ground contact [9], increased net positive work about the ankle [9,10], decreased negative work about the ankle [10] and reduced braking impulse [14–17]. Using ultrasonography, Farris & Raiteri [18] demonstrated that the increase in network about the ankle during acceleration was produced by plantar flexor muscles contraction, while ankle angle and the muscle-tendon unit length remained relatively consistent during mid-stance. Joint mechanics linked to torque production about the ankle during constant speed and accelerative walking are therefore very different, and further examination is needed to clarify how torque production is altered over a range of accelerations.

**Figure 1.**
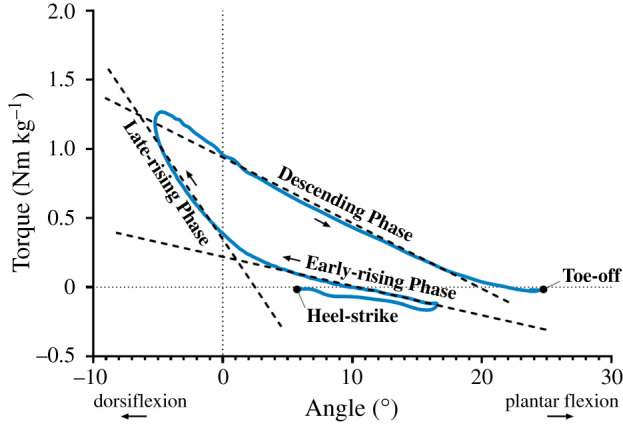
Example of ankle torque–angle relationship from a single participant during constant speed walking, averaged over multiple steps, describing the early-rising phase, late-rising phase and the descending phase.

To explore how torque is produced about the ankle, the torque–angle relationship during stance has previously been examined during constant speed walking [12,19]. As can be seen in figure 1, distinct regions of the ankle torque–angle curve have been described by three linear relationships during stance [19,20], identified as the early-rising phase, late-rising phase and a descending phase [19], while the area within the torque/angle curve represents the network produced about the ankle during stance. During constant speed walking, the early-rising and late-rising phases both include increasing plantar flexor torque paired with increasing ankle dorsiflexion (figure 1); however, the slope of the linear regression fit during late-rising phase is greater than the early-rising phase due to plantar flexor torque increasing more relative to ankle dorsiflexion (figure 1). The descending phase then presents a declining plantar flexor torque and increasing plantar flexion angle.

Previous studies examining increasing constant walking speeds have identified that the gradient of the early-rising phase and the descending phase remained unchanged, while the gradient of the late-rising phase increased until this phase was produced by increasing plantar flexor torque with no change in ankle angle [12,20,21]. Farris & Raiteri [18] simulated accelerative gait on a treadmill and found that compared with constant speed walking, accelerative gait involved a shift in the late rising phase, similar to increasing constant speed (increase in ankle plantar flexor moment with no change in ankle angle). Thus, examining changes within phases demonstrated that accelerative gait has a very different control strategy for coordinating ankle torque and angle compared with preferred constant speed walking. Unfortunately, Farris and Raiteri [18] only examined walking at a single acceleration, and therefore it is unknown how the torque–angle relationship changes as acceleration increases. Understanding how joint-level coordination and timing of torque production changes about the ankle to produce a range of accelerations will greatly assist with understanding how work about the ankle is performed.

Optimizing timing of ankle torque to produce network is a key aspect of designing ankle prosthetics and assistive exoskeletons. The primary purpose of powered assistive devices is to mimic gait in such a way as to either reduce the metabolic cost for locomotion [2,3,22] or assist with minimizing gait abnormalities [4,23]. Adjustments to ankle joint control strategies needed for acceleration may be of particular interest to designers of ankle exoskeletal or prosthetic assistive devices that use ankle position (angle) as a control parameter for torque production [23–27]. As such, torque–angle relationships could be employed as an intuitive method for adjusting control strategies of assistive devices [20]. Research using assistive devices has demonstrated that functional gait requires torque, work and power to be produced at the optimal time and amplitude [25,28,29]. Furthermore, parameters such as peak torque, timing of peak torque, timing of torque onset and rate of torque development have all been shown to strongly influence metabolic cost during constant speed walking with exoskeletal devices [22,28,30]. Humans are capable of adapting their joint coordination strategy if assistive devices are not ideally controlled [25,29,31]; however, this adaptation may not result in gait that is beneficial to the user. Understanding how ankle torque–angle relationships are adapted during healthy accelerative gait could have significant implications for the development of such assistive devices.

This study aimed to explore how torque and work about the ankle is produced as walking acceleration increases from no acceleration to maximal acceleration. We hypothesized that as acceleration increases, increased work about the ankle would not be due to solely ramping up plantar flexion torque, and instead would be a product of adjustments to the relative timing of ankle torque and angular displacement. We additionally hypothesized that as acceleration increases, there would be a proportional increase in the gradient of the ankle torque–angle relationship during the late-rising phase.

## 2. Methods

### 2.1. Participants and protocol

Fifteen participants (nine male and six female, age 27 ± 4 years, height 175 ± 9 cm, mass 70 ± 11 kg) gave written informed consent to participate in this study, which was approved by the Sport and Health Sciences Ethics Committee at the University of Exeter (180509/A/01). Participants attended the biomechanics laboratory at the University of Exeter on one occasion and performed barefoot walking. Participants were asked to walk without any acceleration (constant speed walking), henceforth referred to as no acceleration walking, as well as a self-judged low, moderate and maximal acceleration. For the no acceleration condition, participants were told to walk at a comfortable speed that was slightly slower than their preferred walking speed. Once this had been practised, walking speed when approaching an in-ground force plate was controlled for all conditions by a metronome that matched the participants' step frequency during their no acceleration walking condition. Participants practised accelerative gait by performing a maximal acceleration, which helped identify the range between 0 and 100% acceleration, after which they practised moderate and low walking accelerations. Participants performed a self-selected warm-up and active motion capture markers (LED) were placed on their body. Once placed, participants performed no, low, moderate and maximal accelerative walking conditions in a block randomized order. During acceleration trials, participants performed the first acceleration step on the force plate and were instructed to push-off with the desired acceleration, which was maintained for three steps without initiating a running gait. Participants performed a total of eight successful walking trials per condition, where their right foot landed on the in-ground force plate, and they were not targeting the plate.

### 2.2. Data collection and processing

Three-dimensional motion capture data (200 Hz) were collected using four scanning units (Codamotion, Rothley, UK) that each housed three motion sensors, via ODIN software (Codamotion, Rothley, United Kingdom). Force data (1000 Hz) for the right leg were obtained from a single in-ground force plate (BP400600HF; AMTI, Massachusetts) and logged synchronously using ODIN. Thirty-two active infrared LED markers were placed on the right leg and foot. Foot markers were placed in accordance with the IOR multi-segment foot marker set [32], with additional markers on the distal calcaneus, medial and lateral malleoli, medial and lateral knee joint-line, left and right anterior superior-iliac crest and posterior superior-iliac crest, and clusters of four markers placed midway along the lateral side of the right shank and thigh. Motion capture and force plate data were exported to Visual3D (C-Motion, Maryland, USA) for processing. Using a static trial, a generic rigid-body model (pelvis, right thigh, right shank and right multi-segment foot) defined according to Visual3D's standard geometries and scaled based on segment endpoints and body mass. A second-order two-way low-pass Butterworth filter was used to filter motion capture (cut-off = 10 Hz) and ground reaction force (GRF) data (cut-off = 25 Hz). The scaled model was combined with filtered motion capture and force plate data to obtain six degree of freedom (d.f.) segment kinematics and inverse dynamic ankle joint torques. The ankle joint was defined with six d.f. between rigid-bodies of the shank and rearfoot, with a three-segment foot defined as per the IOR model [32]. Defining the ankle joint in this way has been shown to provide improved estimation of joint angle changes [33], which has important implications for the ankle torque–angle relationship, especially compared with previous research that used a single rigid foot segment [12,19,34,35]. Neutral ankle angle (0°) was defined as the ankle position during quiet standing with plantar flexion defined as positive. Data were exported to Matlab (MathWorks, Natick, MA, USA) and normalized to 501 points over stance. Torque was normalized to body mass and then pelvis centre of mass (CoM) position, GRF, spatio-temporal, kinematic and kinetic outcome measures were calculated.

Ankle plantar flexion joint angle during stance was calculated and its time derivative (joint velocity) was multiplied by plantar flexion joint torque to calculate ankle joint power. Net ankle joint work was calculated by integrating net ankle joint plantar-dorsi flexion power values during stance; positive ankle joint work was calculated by integrating positive ankle power values during stance; negative joint work was calculated by integrating negative ankle power values during stance. Ankle angular impulse was calculated by integrating ankle torque, and average ankle rate of torque development was obtained by dividing peak ankle torque by the time between start of plantar flexion ankle torque (zero ankle torque) and peak ankle torque. Because we only had values for stance, plantar flexion torque onset timing as a per cent of stride was calculated to enable comparison with previous research, as end of stance timing (% stride) is relatively similar between constant speed and accelerative walking [10]. Toe-off was estimated to occur at 60% stride, therefore: stride time = stance time + (stance time × ⅔).

Due to only having markers on the pelvis and right leg (missing data of the left leg and trunk), and a restricted motion capture volume that only obtained stance of the right leg, whole-body acceleration over one stride could not be accurately determined using kinematics. Therefore, per cent of maximal acceleration was used as a surrogate measure for acceleration and was calculated by dividing individual net accelerative GRF impulse for each trial by the maximal net accelerative GRF impulse achieved for each participant across all their trials. By computing this for each trial, we were able to analyse the data with acceleration as an independent variable on a continuous scale, rather than using categorical experimental conditions (i.e. no, low, medium and high acceleration), where constant speed gait is simply zero (−10 to 10% acceleration. It should be noted that because we did not have a second force plate, impulse and accelerations are for the right leg only, and do not account for left leg push-off and braking that would occur at the start and end of stance.

**Figure 2.**
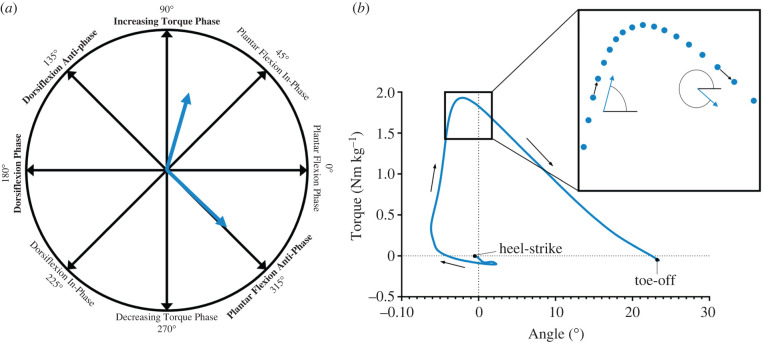
Vector coding methods used to define the 360° angle of the ankle torque–angle relationship between each data point. Example ankle torque–angle curve demonstrates how the 360° angle between data points is identified and correlates to the four key phases identified in this study (increasing torque phase, dorsiflexion phase, dorsiflexion anti-phase and plantar flexion anti-phase).

Initially, we set out to classify three linear relationships within the ankle torque–angle relationship (early-rising, late-rising and descending phase) using the same method as Crenna & Frigo [19]. However, this proved impractical as clear onset of the late-rising phase was very difficult to identify in individual trials. Therefore, we employed vector coding to analyse the relationship between ankle torque and angle. In-depth detail of the rationale for vector coding can be found in electronic supplementary material, file S1, along with averaged torque–angle relationships for each participant. Vector coding is commonly used as a method to examine coordination variability [36,37]; however, this method also performs well at quantifying how two measures are increasing or decreasing in relation to one another. In this study, we used vector coding methods on ankle torque (vertical-axis) and ankle angle (horizontal-axis) and to examine the ankle torque–angle relationship (figure 2). Time-normalized torque and angle magnitudes were normalized to the maximal ankle torque or ankle angle (plantar flexion) for each participant across all their trials [38]. The relationship between the two measures was classified based on their 360° proximity to reference angles representing predefined coordination patterns (figure 2): 90° increasing torque phase (increasing ankle plantar flexion torque with no change in ankle angle); 135° dorsiflexion anti-phase (ankle plantar flexion torque and dorsiflexion angle are increasing proportionally); 180° dorsiflexion phase (increasing dorsiflexion angle with no change in ankle plantar flexion torque); and 315° plantar flexion anti-phase (decreasing ankle plantar flexion torque while ankle plantar flexion angle is increasing proportionally). Plantar flexion was defined as positive, and therefore, simultaneously increasing dorsiflexion angle and plantar flexion torque was defined as dorsiflexion anti-phase (decreasing plantar flexion and increasing ankle torque). It should be noted that this notation is less clear when plantar flexion torque and dorsiflexion torque both occur during the movement. However, this limitation did not impact the results of this study, as dorsiflexion torque was not produced except very briefly during initial ground contact (heel-strike to foot flat). The 360° angle produced between consecutive ankle torque–angle data points was obtained and rounded to the nearest whole integer (figure 2). To visualize our results on a continuous acceleration scale, a three-dimensional frequency graph was created by summing the total number of instances that each 360° angle occurred. To highlight the effect of acceleration on the fraction of stance time spent close to different phases, groupings within the frequency plot were identified by visual inspection.

### 2.3. Statistical analysis

**Figure 3.**
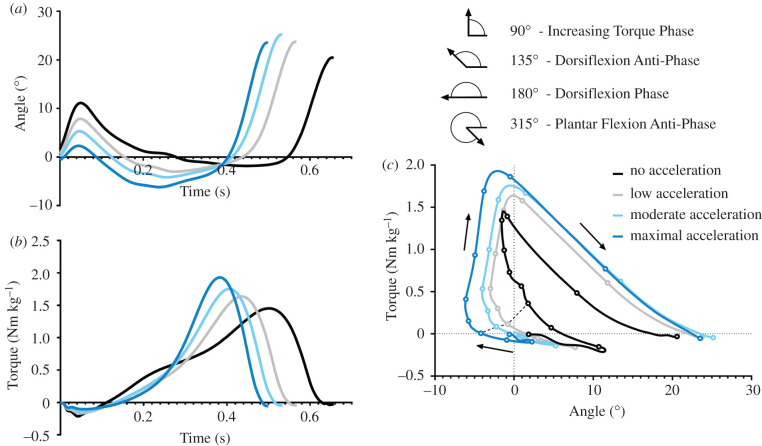
Mean ankle joint angle (*a*) and torque (*b*) for each walking condition (plantar flexion = positive). Dots on ankle torque–angle curve (*c*) represent 10% portions of stance with a dashed line connected between the dots corresponding to 30% of stance. Heel-strike to toe-off occurs in a clockwise direction as indicated by arrows. Vector coding angles of the four key phases identified within this study are illustrated.

Due to marker dropout or a horizontal impulse less than −10% maximal acceleration (deceleration), only three no acceleration trials were usable for one participant and five for another. However, because linear mixed modelling was performed on continuous data (% maximal acceleration) and is robust to missing data points, we do not believe this biased the results. All other participants had at least six to eight successful trials per condition (total of 456 successful trials analysed). Linear regression graphs for each outcome measure are presented in electronic supplementary material, file S2 with overall lines of best fit and the average gradient of change reported in table 1 to demonstrate the overall change and provide justification for using linear mixed models (LMMs). LMMs were performed to assess the effect of acceleration on outcome measures on a continuous scale. LMMs were designed such that each outcome variable was analysed with per cent maximal acceleration and average horizontal pelvis CoM velocity as fixed effects, and participant (repeat measures) as a random effect (452 degrees of freedom). Average pelvis velocity was employed as a fixed effect, as ankle work and torque are systematically influenced by walking speed [12]. To examine if there was a significant effect of acceleration on each output variable, a likelihood ratio test was performed between each LMM and their null counterpart (without *acceleration* as a fixed effect). To account for multiple comparisons, Bonferroni *post hoc* analysis was performed, reducing the alpha from 0.05 to 0.003. Raw *p*-values are reported but they were only considered significant if below the adjusted alpha. Acceleration values were input into the LMM as ranging from −0.1 to 1 (−10 to 100% acceleration), therefore LMM estimates for acceleration are interpreted as the estimated change as acceleration increased from 0% acceleration to 100% acceleration. LMM estimate, 95% confidence interval and *p*-values from the likelihood ratio test are presented. Ankle angle, torque and torque–angle relationship group mean graphs for each categorical condition (no, low, medium and maximum acceleration) are presented for ease of viewing the data (figure 3). However, all outcome variable statistics reported in text used the LMM change and therefore describe the change as acceleration increased from 0 to 100% acceleration.

**Table 1.** Key outcome measures are presented using the linear changes as acceleration increases from 0 to 100% maximal acceleration. Linear regression lines of best fit averages, along with linear mixed model (LMM) mean change, 95% confidence interval (CI) and *p*-value are presented. Positive values demonstrate an increase in the outcome measures as acceleration increases, while negative values demonstrate a decrease in the outcome measure as acceleration increases.

outcome measure	linear regression	linear mixed model		
	line of best fit gradient	estimated change due to acceleration	CI (95%)	*p*-value
stance time (s)	−0.158	−0.019	−0.032 to −0.006	0.005
peak accelerative Grf (N)	123	114	100 to 127	<0.001
peak braking GRF (N)	−108	−72	−86 to −58	<0.001
minimum horizontal velocity of the pelvis CoM, relative to velocity at heel-strike (m s^−1^)	0.32	0.22	0.17 to 0.28	<0.001
maximum horizontal velocity of the pelvis CoM, relative to minimum pelvis velocity (m s^−1^)	0.64	0.37	0.32 to 0.472	<0.001
pelvis horizontal CoM position, relative to the anterior border base of support (toe), at the instant of peak torque (mm)	81	72	57 to 88	<0.001
*ankle joint*				
net work (J kg^−1^)	0.35	0.18	0.16 to 0.23	<0.001
positive work (J kg^−1^)	0.28	0.15	0.11 to 0.19	<0.001
negative work (J kg^−1^)	−0.07	−0.04	−0.06 to −0.2	<0.001
maximal torque (Nm kg^−1^)	0.57	0.65	0.58 to 0.73	<0.001
angular impulse (Nm s kg^−1^)	−0.09	0.01	−0.02 to 0.03	0.635
time between heel-strike and onset of positive plantar flexion torque (s)	0.045	0.078	0.060 to 0.096	<0.001
time between heel-strike and onset of positive plantar flexion torque (% stride)	8.6	8.5	6.5 to 10.4	<0.001
time between onset of positive plantar flexion torque and peak plantar flexion torque (s)	−0.160	−0.068	−0.089 to −0.047	<0.001
time between onset of positive plantar flexion torque and peak plantar flexion torque (% stride)	−8.2	6.3	−8.3 to −4.3	<0.001
average rate of torque development (Nm s^−1^)	5.75	4.20	3.44 to 4.98	<0.001
ankle plantar flexion angle at heel-strike (°)	−2.4	−0.6	−2.0 to 0.7	0.359
ankle plantar flexion angle during initial ground contact (°)	−6.7	−4.6	−5.7 to −3.4	<0.001
horizontal distance between ankle joint and pelvis CoM at heel-strike (mm)	9	13	9 to 17	<0.001

## 3. Results

Average pelvis velocity during stance was 1.45 ± 0.13 m s^−1^ for 0% acceleration and linearly increased up to 2.14 ± 0.30 m s^−1^ for 100% acceleration. Statistical analyses reported in table 1 include LMM estimated change as acceleration increased from 0 to 100% maximal acceleration with 95% confidence intervals, adjusted for fixed and random effects within the LMM. For reference, table 1 also includes the mean change of lines of best fit from the linear regression graphs presented within the electronic supplementary material, file S2, to illustrate the model fits.

### 3.1. Ankle torque–angle relationship

**Figure 4.**
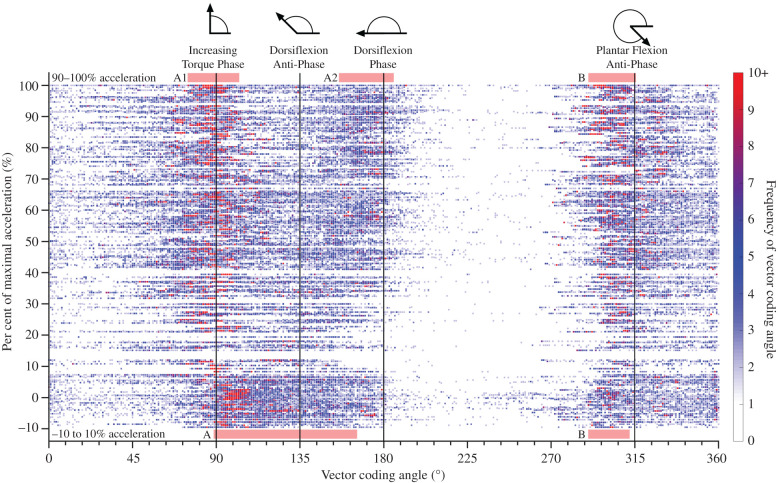
Frequency plot of vector coding angles. Individual trials are plotted along the vertical axis based on per cent of maximal acceleration, thus individual trials can be observed as 456 dotted horizontal lines, which overlap (456 trials), with each dot in the horizontal line representing the frequency of each individual 360° phase angle (horizontal axis) for that trial. Colour intensity values indicate the number of times that an angle is present within the 501 data time points of stance. Thus, red values represent that angle is being performed with high frequency of at least 10 data points during stance. Angle frequency values of 0 are transparent. Groupings (red boxes) were identified between −10 and 10% acceleration (A and B), and 90–100% acceleration (A1, A2 and B). Four key reference phases are depicted as vertical lines: 90° increasing torque phase, 180° dorsiflexion phase, 135° dorsiflexion anti-phase and 315° plantar flexion anti-phase.

The decrease in negative work and subsequent increase in net positive work that occur with increasing acceleration (table 1) can be explored by examining the ankle torque–angle relationship in figure 3*c* and the vector coding frequency graph in figure 4, which illustrates where the torque–angle curve predominantly operated during stance relative to key vector coding phases. In figure 4, groupings of data can be seen to shift as acceleration increased and have been highlighted (red bars) using visual inspection between −10 and 10% acceleration and 90–100% acceleration (maximal acceleration). At −10 to 10% acceleration, Group A (figure 4), was centred slightly to the right of the increasing torque phase (highest density and colour intensity) and extended through dorsiflexion anti-phase, indicating that ankle dorsiflexion was occurring as ankle plantar flexion torque increased. Alternatively, as acceleration increased, Group A split into two distinct groupings (Groups A1 and A2) with a sparse area in between. Group A1 was centred directly over the increasing torque phase (figure 4), indicating that accelerative torque increased with no change in dorsiflexion, regardless of the level of acceleration (figures 3*c* and 4). Alternatively, Group A2 moved toward the dorsiflexion phase as acceleration increased, indicating per cent of stance in passive dorsiflexion increased with acceleration magnitude (figures 3*c* and 4). Finally, Group B is primarily made up from plantar flexion during push-off (figure 3*c*) and remained similar across all accelerations (figures 3*c* and 4).

## 4. Discussion

This study aimed to explore how angle, torque and work about the ankle are produced as walking acceleration increased from 0 to 100% maximal acceleration. We found a clear difference in how torque was produced about the ankle during early and mid-stance as acceleration increased. Primarily, differences about the ankle were found in net, positive and negative work, timing and magnitude of torque production, and the ankle torque–angle relationship morphed from a biphasic group pattern during walking without acceleration, to a triphasic group pattern as walking accelerations increased.

### 4.1. Ankle joint work

When accounting for fixed and random effects as acceleration increased, network about the ankle increased due to greater positive work and reduced negative work (table 1). Negative work about the ankle decreased to near zero as acceleration increased up to maximal (electronic supplementary material, file S2) due to reduced braking force, which subsequently reduced pelvis CoM deceleration during early/mid-stance (table 1). This result supports previous work which also demonstrated that braking impulse decreases as acceleration increases [14–17]. However, the increase in positive work about the ankle was four times greater than the decrease in negative work. Therefore, increased network about the ankle during accelerative walking was probably performed by a greater reliance on plantar flexor muscles to produce a positive ankle work as acceleration increased and contribute to a greater peak accelerative GRF [18], with very limited energy absorption from CoM momentum (reduced negative work). This is opposed to walking without acceleration, where the body's kinetic energy is converted to Achilles tendon strain energy while the plantar flexor's muscle fascicles act isometrically [13].

### 4.2. Ankle torque and timing

The increase in positive and network about the ankle required for acceleration was primarily produced by an increase in peak torque (figure 3*b*). However, when accounting for fixed and random effects as acceleration increased, stance time remained constant, onset of plantar flexor torque was delayed (table 1) and peak torque occurred significantly earlier (figure 3*a*). Therefore, increasing acceleration required a significantly greater average rate of torque development about the ankle. These findings support previous research, where walking acceleration relied upon a delayed onset of lateral gastrocnemius muscle activation, paired with greater activation ramping to a higher maximal magnitude [18]. The delayed onset of plantar flexor torque was probably one factor that enabled a decrease in negative work about the ankle, enabling the ankle to move through dorsiflexion with minimal plantar flexor torque and reduced braking force to maintain a higher pelvis CoM velocity during early mid-stance. Despite an increase in maximal ankle torque as acceleration increased, there was no change in ankle angular impulse, which was probably due to no change in stance time combined with delayed onset of plantar flexor torque. This supports our first hypothesis that an increase in ankle work as acceleration increases would not be due to solely ramping up plantar flexion torque and would also be a product of adjustments to the relative timing of ankle torque and angular displacement.

### 4.3. Ankle torque–angle relationship

This study applied vector coding methods to examine how the ankle torque–angle relationship during stance changed with increasing acceleration (figures 3*c* and 4). Our results indicate that preferred walking without acceleration is biphasic, with two groupings (A and B) emerging in the vector coding frequency graph (figure 4), while previous studies have suggested that preferred constant speed walking is triphasic [19]. When walking without acceleration, Group A had the greatest density and colour intensity of points (spacing and red colour) situated to the right of the increasing torque phase and skewing through the dorsiflexion anti-phase. While vector coding has no time dimension, figure 3*c* demonstrates that Group A was primarily produced during early and mid-stance of walking with no acceleration, where the ankle torque and dorsiflexion angle are increasing proportionally and probably relate to underlying storage of energy within the Achilles tendon [13]. Group B had a relatively narrow spacing of points when walking without acceleration, which was situated closest to the plantar flexion anti-phase (figure 4), with figure 3*c* indicating it is primarily produced during push-off (late stance) and seems well-suited to representation by a linear function (figure 3*c*). When there was no acceleration, Group A spans from dorsiflexion anti-phase to increasing torque phase without an obvious transition point (figures 3*c* and 4), therefore attempting to split the ankle torque–angle curve into two separate linear relationships during early mid-stance (Group A) may be problematic, and a single nonlinear function would be more appropriate. Alternatively, previous studies have shown that as constant walking speed increases, a triphasic pattern emerges, with a clear transition point appearing in the ankle torque–angle relationship between early and mid-stance [12,20,21]. Therefore, when employing vector coding analysis on fast constant speed walking, we predict that three distinct groups will be present. Ultrasound imaging studies that have explored a range of constant walking and running speeds, suggest that the triphasic pattern at fast speeds is a product of plantar flexor muscle fascicles concentrically contracting to perform greater positive work at higher speeds, instead of acting isometrically [39].

As acceleration increased, a triphasic pattern of three separate groups (A1, A2 and B) emerged (figure 4). Group A1 was centred directly on top of the increasing torque phase and did not change as acceleration increased (figure 4). Group A2 was situated between dorsiflexion anti-phase and dorsiflexion phase but increasingly shifted toward the dorsiflexion phase as acceleration increased (figure 4). Finally, Group B was centred close to the plantar flexion anti-phase and did not appear to change as acceleration increased (figure 4). The additional group was a product of the Group A splitting into two distinct groups (A1 and A2) as acceleration increased (figure 4). Group A2 (figure 4) represents ankle dorsiflexion with minimal plantar flexor torque as acceleration increased, which primarily occurred during early stance (figure 3*c*). Group A1 (figure 4) represents increasing plantar flexion torque with little change in ankle angle, which was primarily occurring during mid-stance following a clear transition period (figure 3*c*). Interestingly, Group A1 and A2 form in very different ways. As acceleration increased, Group A2 migrates toward the dorsiflexion phase, while Group A1 is centred nearly perfectly over the increasing torque phase for all accelerations (figure 4). This contradicts our second hypothesis, which suggested there would be a smooth shift in gradient of the ankle torque–angle relationship during mid-stance, as walking acceleration increased from 0 to 100% maximal walking acceleration.

Therefore, during mid-stance, torque is probably produced similarly between fast constant speed walking [12,20,21] and accelerative walking (figures 3*c* and 4), observing an increase in torque with minimal or no change in dorsiflexion angle. However, similarities are not present during early stance, where fast constant speed walking is produced with an increase in torque and dorsiflexion angle [12,20,21], while accelerative gait is performed with increasingly passive dorsiflexion and reduced plantar flexion torque as acceleration increases (figures 3*c* and 4). As such, fast constant speed walking is probably produced by a combination of storing energy within the tendon during early stance, while muscles act isometrically (increasing torque combined with ankle dorsiflexion angle change) [13], paired with muscles contracting to perform network during mid-stance (increased torque with minimal angle change) [39]. Alternatively, very little energy is likely to be stored in the Achilles tendon during early stance of accelerative gait, as dorsiflexion occurs with minimal plantar flexion torque, hence the delay in muscle activation found previously by Farris & Raiteri [18]. Instead, most of the work about the ankle must be performed by muscle fascicles actively shortening during mid-late stance. Thus, joint mechanics linked to generation of ankle torque and work are fundamentally different between all constant walking speeds and accelerative walking.

An additional question therefore presented itself during data analysis: what mechanisms exist to balance the external and internal forces about the ankle as acceleration increases (figure 3*c*), facilitating increasing ankle plantar flexor torque with no change in ankle angle, irrespective of the magnitude of acceleration? As acceleration increased, minimum pelvis CoM velocity during early/mid-stance (relative to pelvis CoM velocity at heel-strike) also increased due to reduced braking forces (table 1). This maintains a greater CoM angular momentum over the stance leg (considering an inverted pendulum model of stance) which is in opposition to the large plantar flexor torque produced during mid-stance when accelerating. Both minimum pelvis CoM velocity and ankle plantar flexor torque increased as acceleration increased (electronic supplementary material, file S2), which may act to balance each other and produce little or no ankle rotation during mid-stance (figure 3*c*). However, this results in the pelvis horizontal CoM position at peak ankle torque moving further outside of the base of support as acceleration increases (table 1). Pushing the CoM further outside the base of support could have implications for risk of falling in clinical populations [40], making changing walking speed or initiating gait potentially more hazardous than constant speed walking.

### 4.4. Practical applications

Understanding mechanisms for how the body performs accelerative walking has widespread applications in foundational understanding of human movement, clinical gait biomechanics and the development of assistive devices. Our study has demonstrated clear changes to the ankle torque–angle relationship as acceleration increases from 0 to 100% maximal acceleration. Such changes need to be mimicked by control schemes of assistive wearable exoskeletons to avoid hindering the wearer as they try to accelerate. Our results demonstrate that during accelerative steps, a powered exoskeletal or prosthetic device needs to rotate near passively through dorsiflexion during early stance, which requires assistive plantar flexor torque onset to be delayed compared with constant speed walking. Torque magnitude must then rapidly increase while maintaining a fixed ankle position. This results in a very different torque–angle relationship compared with what is currently being implemented by assistive ankle exoskeletons during constant speed walking [25]. Previous research has identified substantial changes in net metabolic cost when altering torque onset timing from assistive devices by as little as 3–10% of stride [30,41]. We estimated that onset delay relative to stride time increased by 8.5% as acceleration increased from 0 to 100% (table 1), and therefore onset timing will probably play a crucial role in altering coordination strategies of assistive devices to produce accelerations that do not negatively alter gait. Changes in timing of torque onset can be examined relative to per cent of the gait cycle, represented by dots spaced every 10% of stance within the ankle torque–angle relationship (figure 3*c*). Dots connected between conditions by a dashed line represent 30% of the gait cycle and can be seen to have reduced torque and increased ankle dorsiflexion angle as acceleration increases. Using ankle torque–angle relationships as input for exoskeletal control systems: torque, angle, work and event timings relative to per cent gait cycle (stance/stride) may be altered.

Using the torque–angle data presented here, accelerative steps produced using torque or position-based control schemes could be parametrized for powered devices [23–26]. Increasing the maximal power and torque that must be produced by powered assistive devices comes at a cost that may require larger and heavier motors and batteries. However, exoskeletal devices could alternatively maintain the same level of torque as constant speed walking and instead depend on muscles of the leg to produce the additional torque required for acceleration. In this way, only the timing of plantar flexor torque onset and average rate of torque development need to be adjusted. Unfortunately, passive devices cannot contribute net positive work and must rely on elastic energy stored during ankle dorsiflexion during early and mid-stance to produce force [2]. Therefore, while microprocessors and clutch mechanisms could be harnessed to alter timing of torque production of passive devices and avoid impeding acceleration, assisting acceleration by altering the rate of torque development to match coordination strategies of both constant speed and accelerative walking remains infeasible with current designs.

To facilitate altered coordination strategies during accelerative walking, devices need to detect the intent to perform an accelerative step. While all accelerative gait appears triphasic in nature, the magnitude of these changes (maximal torque, delay of torque onset and rate of torque development) shift gradually as acceleration increases. When accounting for fixed and random effects within the LMM as walking acceleration increases, ankle plantar flexion during initial ground contact was reduced by 4.6° (figure 3*a*) and ankle position at heel-strike was 13 mm closer to the CoM, while ankle angle at heel-strike was not statistically different (table 1). Identification of these variables during walking could be obtained using angular encoders during initial ground contact, providing an indication not only of intent to accelerate but potentially also the magnitude of desired acceleration. It should be noted that this study only examined the first accelerative stride following constant speed walking, due to availability of a single force plate. Future research may need to examine if accelerative walking over several consecutive strides or from a standing start (gait initiation), present with similar results to this study. Additionally, examination of muscle contractile mechanics during accelerations with and without assistive devices will be crucial to understanding how muscles contribute to altered coordination strategies during accelerative gait [18,42,43]

Pushing the CoM further outside of the base of support during accelerations could potentially result in an increased risk of falling in both physically disabled persons that require assistive devices, as well as elderly populations. Elderly populations have a higher risk of falls due to reduced reaction speed [44] and muscular strength [40]. Subsequently, they are less able to recover from tripping or perturbations [45]. Older individuals also have high spatio-temporal variability during gait initiation [46] and have reduced centre of pressure movements [47], especially during dual tasks for elderly fallers who significantly reduce their anterior displacement of the CoM during gait initiation [47]. High spatio-temporal variability and reduced centre of pressure movement during gait initiation may be a result of trying to maintain the CoM within the base of support but struggling to do so. Further research examining torque production about the ankle and position of the CoM during accelerative walking is needed to understand how elderly people perform accelerative walking movements (gait initiation, accelerative gait changes, turning accelerations) which may be important to help reduce the risk of falling.

## 5. Conclusion

We have demonstrated that at the ankle, accelerative gait is produced with increased average rate of torque development, increased maximal torque and no change in ankle angular impulse despite an increase in ankle network, due to no change in contact time and increasingly delayed onset of plantar flexor torque as acceleration increases. We have employed vector coding in a novel application to examine how joint mechanics to produce torque and work about the ankle change as walk acceleration increases. We found that early stance dorsiflexion occurs with reduced or no resistance as acceleration increases, and mid-stance ankle plantar flexor torque increased with minimal ankle angle change, irrespective of the magnitude of acceleration. As such, development of assistive devices needs to account for these changes during walking to facilitate functional accelerative gait.

## Data Availability

Time normalized data (501 points) of time, angle, moment, work, GRF and position of pelvis and foot for all individual trials has been included within electronic supplementary material, file S3. Electronic supplementary material, data include time normalized (501 points) outcome measures of stance time, ankle angle, ankle moment, ankle work, GRF force, position of the pelvis and position of the ankle for each trial of each individual. The data are provided in electronic supplementary material [48].
